# Supplementary figures and images for: Intestinal delta-6-desaturase activity determines host range for Toxoplasma sexual reproduction
Source: PLoS Biol. 2019 Aug 20;17(8):e3000364. doi: 10.1371/journal.pbio.3000364 (PMC6701743; doi:10.1371/journal.pbio.3000364)

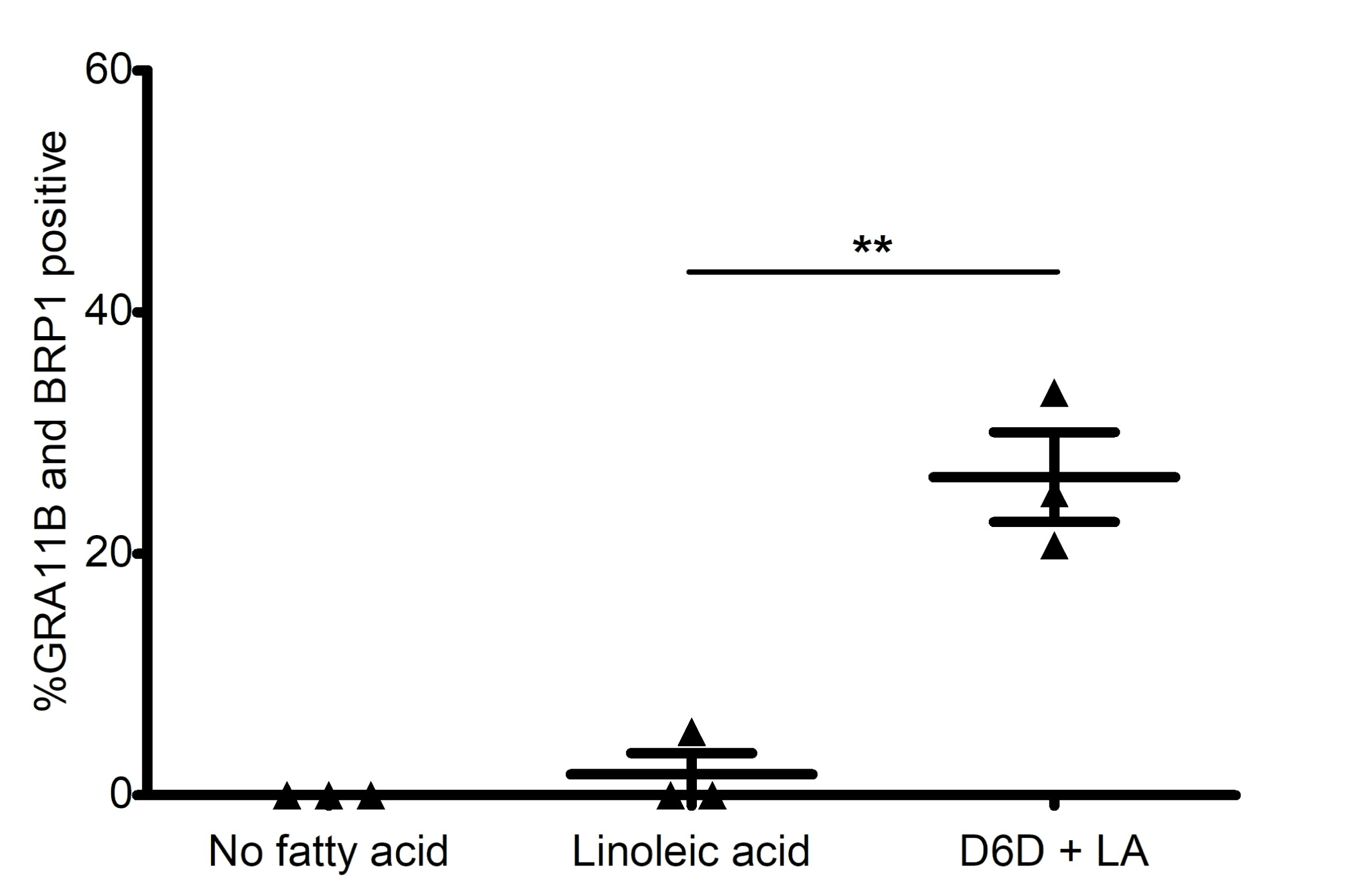

Supplement: S1 Fig — Mouse intestinal organoids were disassociated by trypsin, and the individual cells were grown on glass slides. Slides were divided into three different groups: not supplemented with fatty acids or SC-26196 inhibitor, supplemented with 200 μM linoleic acid, or supplemented with 200 μM linoleic acid plus 20 μM SC-26196 (“D6D +LA”). Monolayers were infected with T. gondii ME49 bradyzoites purified from brains of chronic infected mice. At 5 days post infection, monolayers were stained for GRA11B, BRP1, and DAPI. Total number of parasitophorous vacuoles were counted by positive DAPI staining and confirmed by morphology with DIC. The percentage of vacuoles positive for GRA11B and BRP1 out of the total vacuoles was determined. Three biological replicates were counted, and on average, 26% of the total vacuoles were positive for GRA11B and BRP1 in the linoleic acid–supplemented monolayers with the addition of SC-26196. **p-value = 0.0039 with N = 3 by two-tailed unpaired t test. BRP1, bradyzoite rhoptry protein-1; DIC, differential interference contrast; GRA11B, dense granule protein 11B. (TIF) [file pbio.3000364.s001.tif]

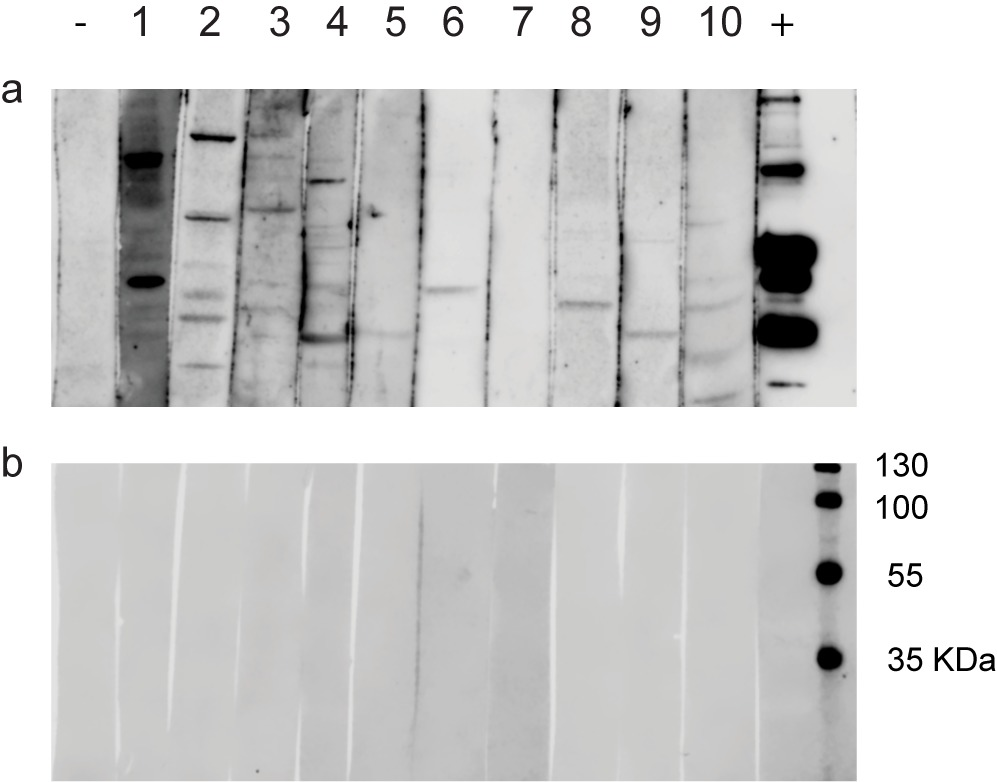

Supplement: S2 Fig — Oocysts collected from SC-26196-treated mice were sporulated and injected intraperitonially into C57BL/6 mice. Serum was collected by terminal bleed at either 22 days (1 and 2), 28 days (3 and 4), or 3 months (5–10) post infection. Serum from uninfected mouse was used as a negative control (−). The positive control (+) was serum from a mouse infected for 26 days with cat oocysts. Serum was incubated with nitrocellulose blotted with ME49 tachyzoite lysate. Panel (a) is chemiluminescence, and (b) is a 700-nm image showing the individual lanes and the protein ladder. (TIF) [file pbio.3000364.s002.tif]

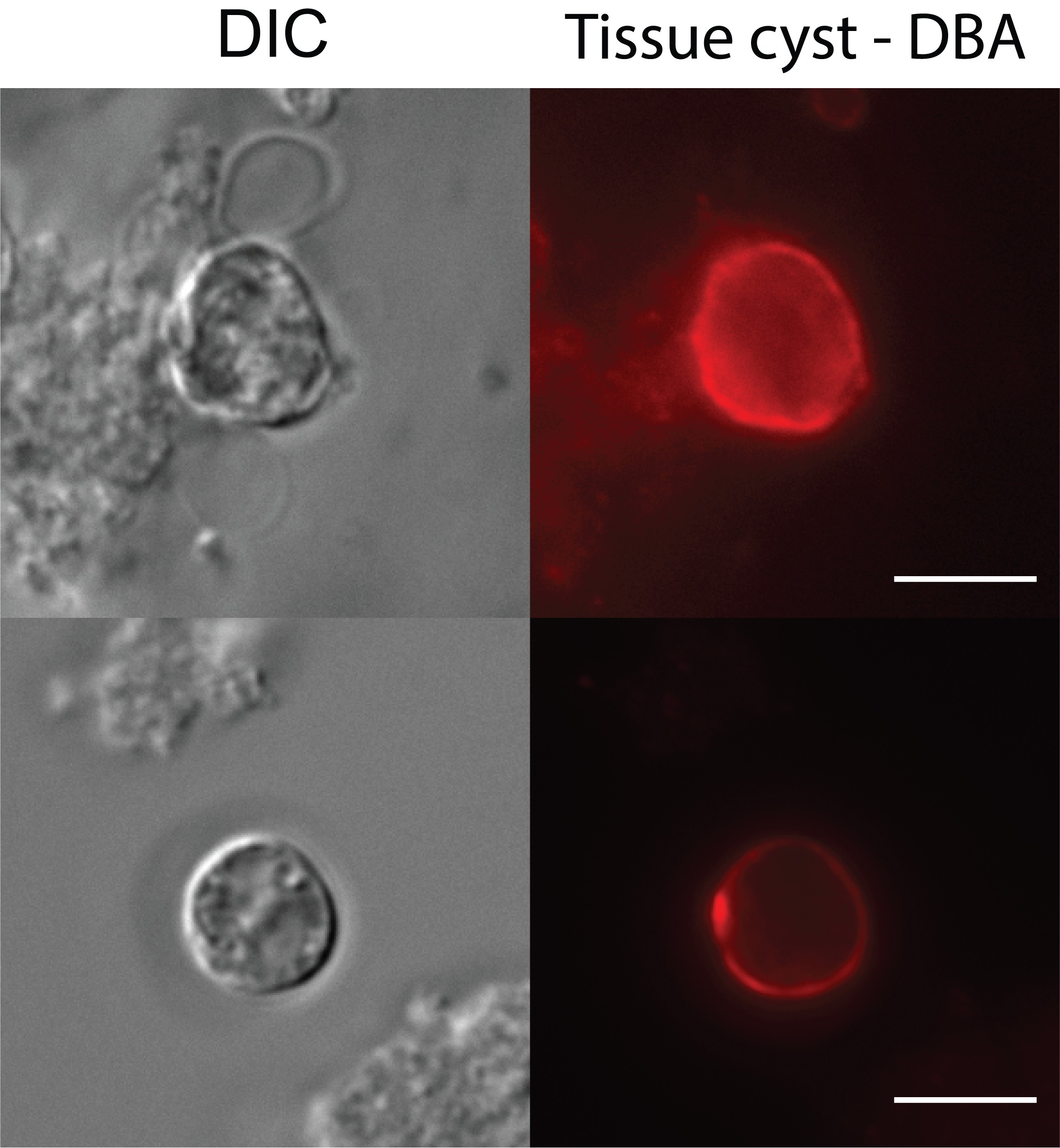

Supplement: S3 Fig — Twenty-eight days post infection with oocysts, mouse brains were removed, homogenized, and stained for T. gondii cysts by DBA (red). All panels are 20 μm2 with a 5-μm white size bar in the lower right corner. DBA, D. biflorus agglutinin. (TIF) [file pbio.3000364.s003.tif]

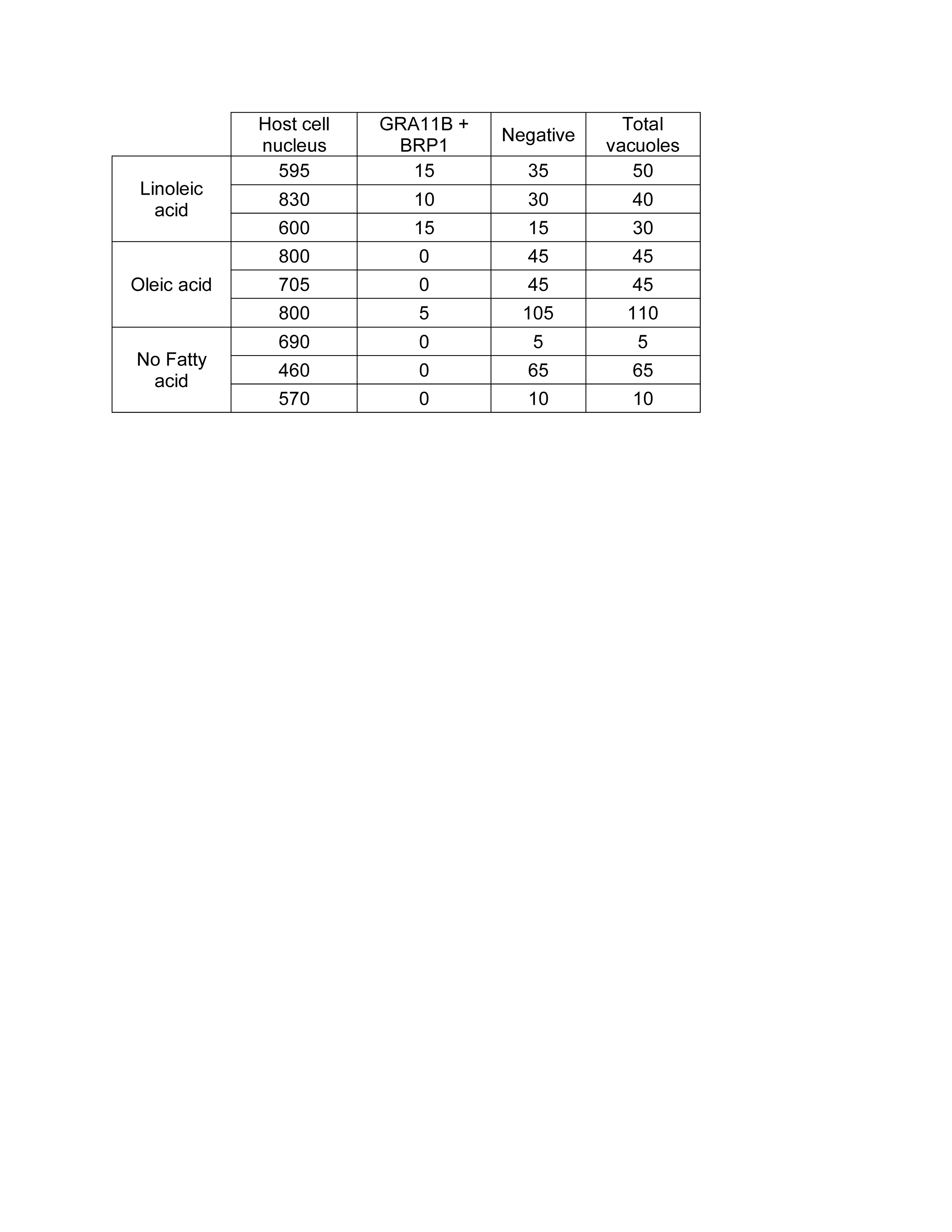

Supplement: S1 Data — Quantification of GRA11B and BRP1 double-positive vacuoles in cat tissue culture. Cat intestinal monolayers were divided into three different groups: not supplemented with fatty acid, supplemented with 200 μM oleic acid, or supplemented with 200 μM linoleic acid (left column). Monolayers were infected with T. gondii ME49 bradyzoites purified from brains of chronic infected mice at a 1:10 MOI. Five days after infection, staining was performed for GRA11B and BRP1 along with DAPI. For each random field, the number of host cell nuclei was counted along with GRA11B/BRP1 double-positive and negative vacuoles. For each biological replicate, five different technical replicates were counted, and the average value is presented. Each line of the table is an independent experiment, three biological replicates. BRP1, bradyzoite rhoptry protein-1; GRA11B, dense granule protein 11B; MOI, multiplicity of infection. (TIF) [file pbio.3000364.s004.tif]

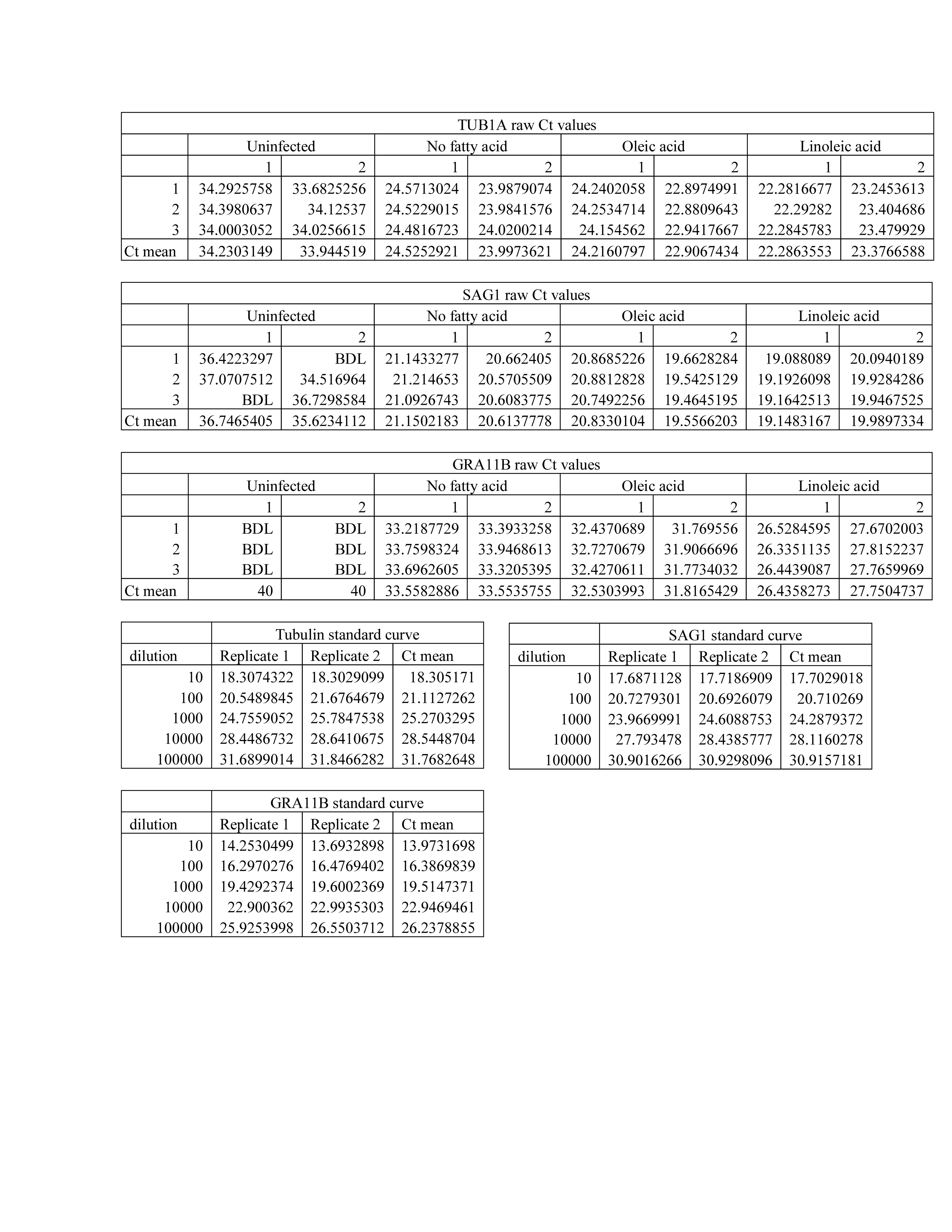

Supplement: S2 Data — Raw Ct values of TUB1A, SAG1, and GRA11B from the cDNA of cat intestinal monolayers samples using TUB1A as the normalizer for target gene expression. Wells with multiple melt curve temperatures, indicating off-target products, were excluded (“NA”). Samples below the detection limit of 40 cycles are labeled BDL. BRP1, bradyzoite rhoptry protein-1; GRA11B, dense granule protein 11B; SAG1, surface antigen 1; TUB1A, tubulin 1A. (TIF) [file pbio.3000364.s005.tif]

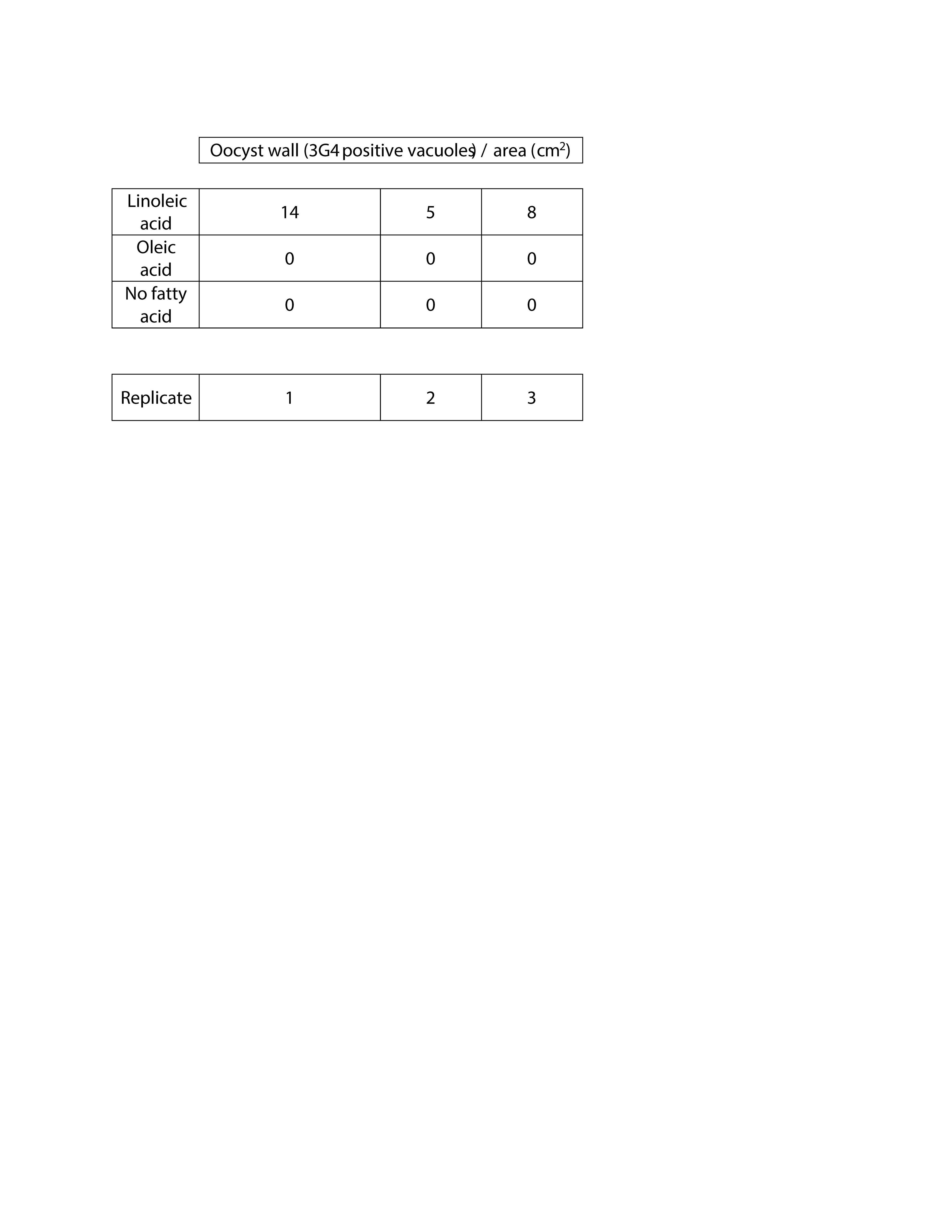

Supplement: S3 Data — Quantification of vacuoles positive with the oocyst wall antibody 3G4 in cat tissue culture. Cat intestinal monolayers were divided into three different groups: not supplemented with fatty acid, supplemented with 200 μM oleic acid, or supplemented with 200 μM linoleic acid (left column). Monolayers were infected with T. gondii ME49 bradyzoites purified from brains of chronic infected mice at a 1:10 MOI. Seven days after infection, staining for oocyst wall antigen was performed with 3G4 antibody. For each 1-cm2 well, the total number of 3G4 positive vacuoles was counted. Each column of the table is an independent experiment, three biological replicates. MOI, multiplicity of infection. (TIF) [file pbio.3000364.s006.tif]

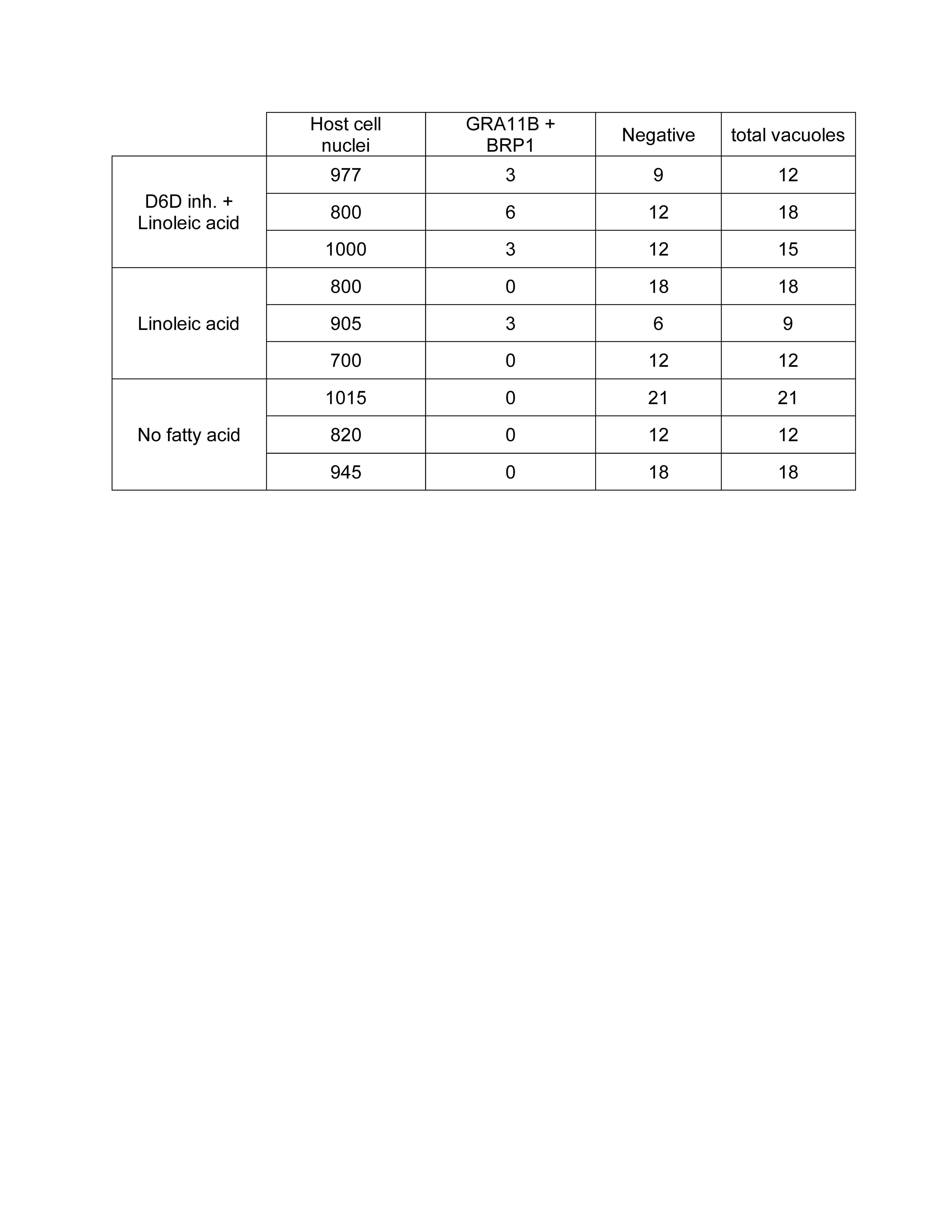

Supplement: S4 Data — Quantification of GRA11B and BRP1 double-positive vacuoles in mouse tissue culture. Mouse intestinal monolayers were divided into three different groups: not supplemented with fatty acids or SC-26196 inhibitor (no fatty acid), supplemented with 200 μM linoleic acid, or supplemented with 200 μM linoleic acid plus the addition of 20 μM SC-26196 (“D6D inh.”). Monolayers were infected with T. gondii ME49 bradyzoites purified from brains of chronic infected mice at a 1:10 MOI. Five days after infection, staining was performed for GRA11B and BRP1 along with DAPI. For each random field, the number of host cell nuclei was counted along with GRA11B/BRP1 double-positive and negative vacuoles. For each biological replicate, three different technical replicates were counted, and the average value is presented. Each line of the table is an independent experiment, three biological replicates. BRP1, bradyzoite rhoptry protein-1; GRA11B, dense granule protein 11B; MOI, multiplicity of infection. (TIF) [file pbio.3000364.s007.tif]

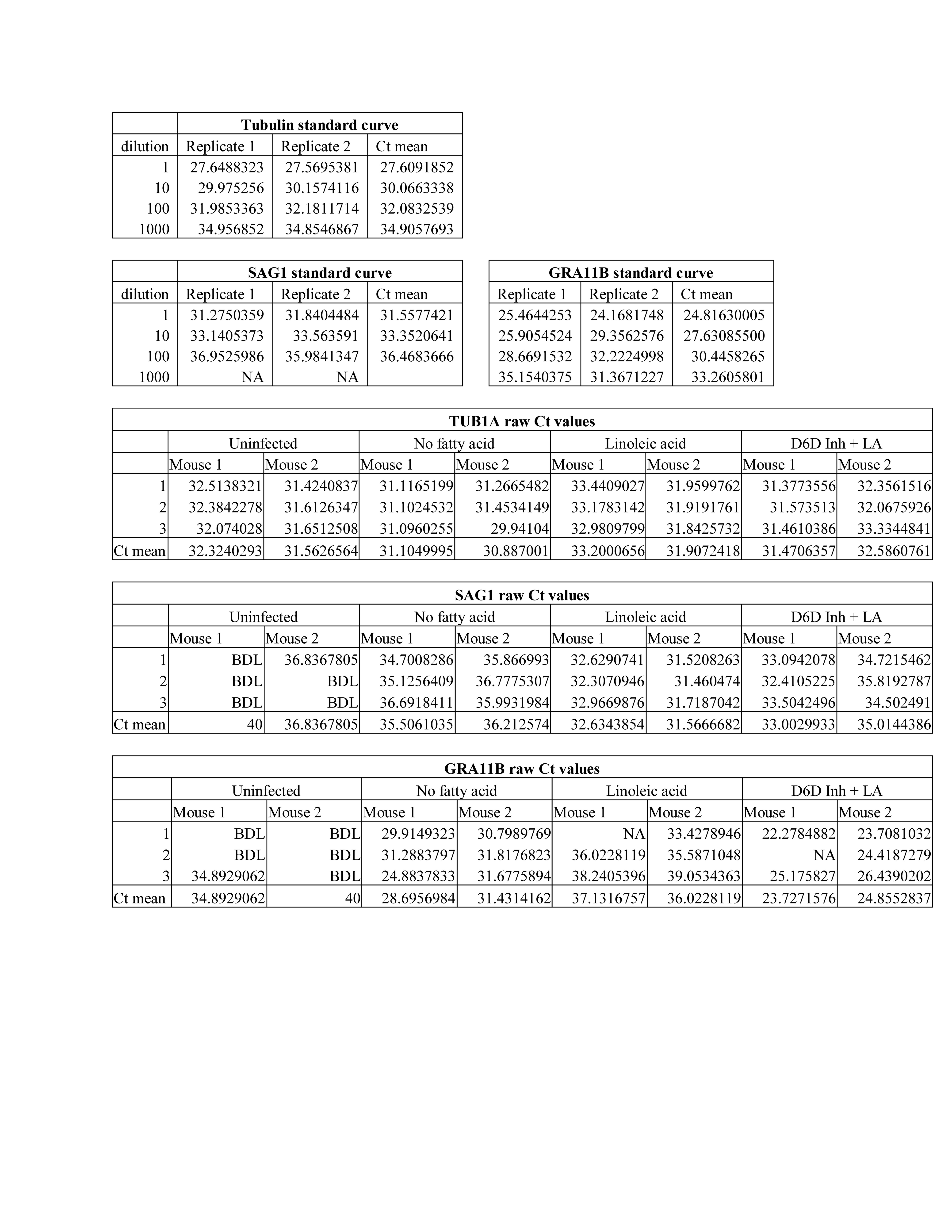

Supplement: S5 Data — Raw Ct values of the TUB1A, SAG1, and GRA11B standard curves on a dilution series of genomic DNA from tachyzoites and raw Ct values of TUB1A, SAG1, and GRA11B from the cDNA of homogenized mouse ileum samples using TUB1A as the normalizer for target gene expression. Wells with multiple melt curve temperatures, indicating off-target products, were excluded (“NA”). Samples below the detection limit of 40 cycles are labeled BDL. BRP1, bradyzoite rhoptry protein-1; GRA11B, dense granule protein 11B; SAG1, surface antigen 1; TUB1A, tubulin 1A. (TIF) [file pbio.3000364.s008.tif]

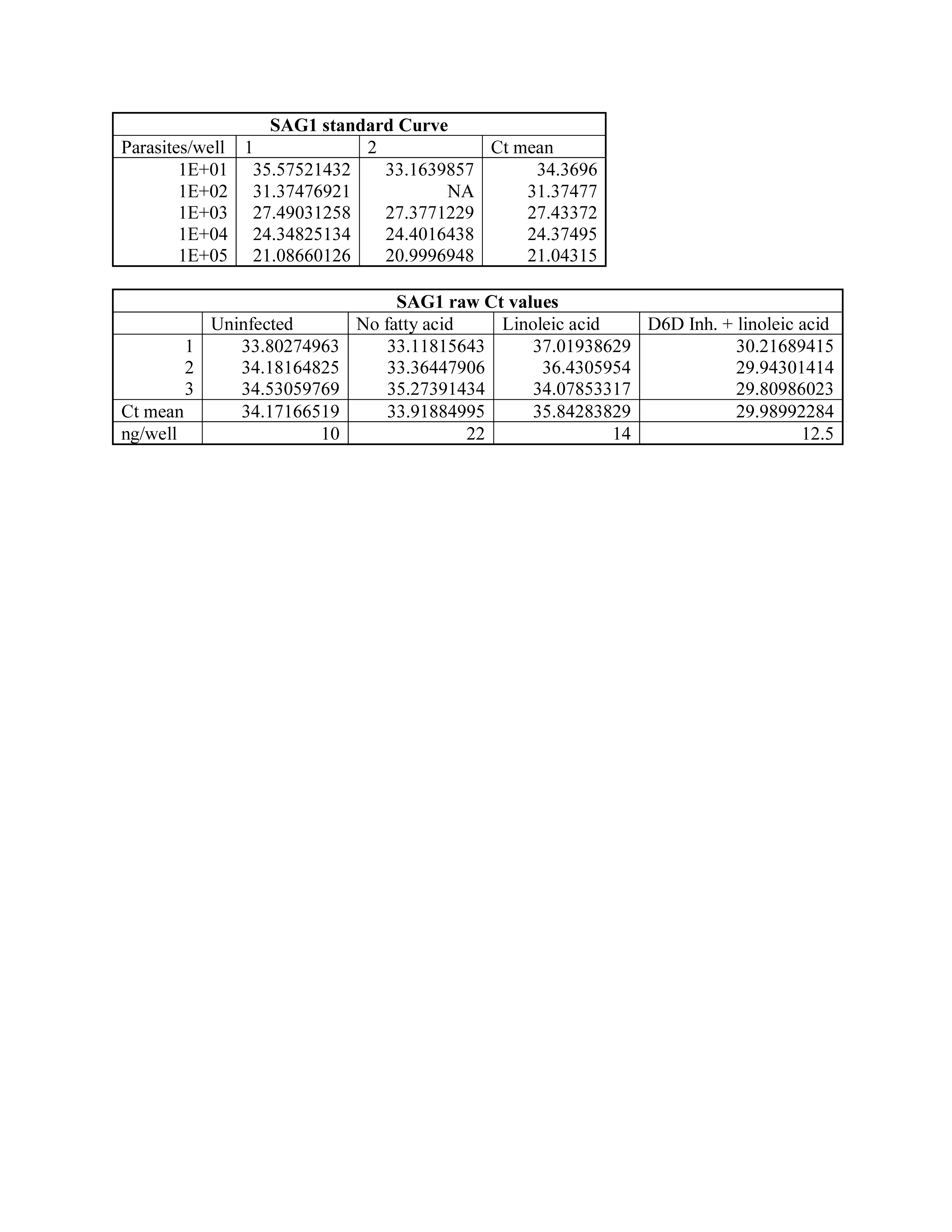

Supplement: S6 Data — Raw Ct values of the SAG1 standard curve on a dilution series of genomic DNA with known parasite quantity and raw Ct values of SAG1 amplification from genomic DNA of unknown fecal samples using ng quantity input as the normalizer. Wells with multiple melt curve temperatures, indicating off-target products, were excluded (“NA”). SAG1, surface antigen 1. (TIF) [file pbio.3000364.s009.tif]

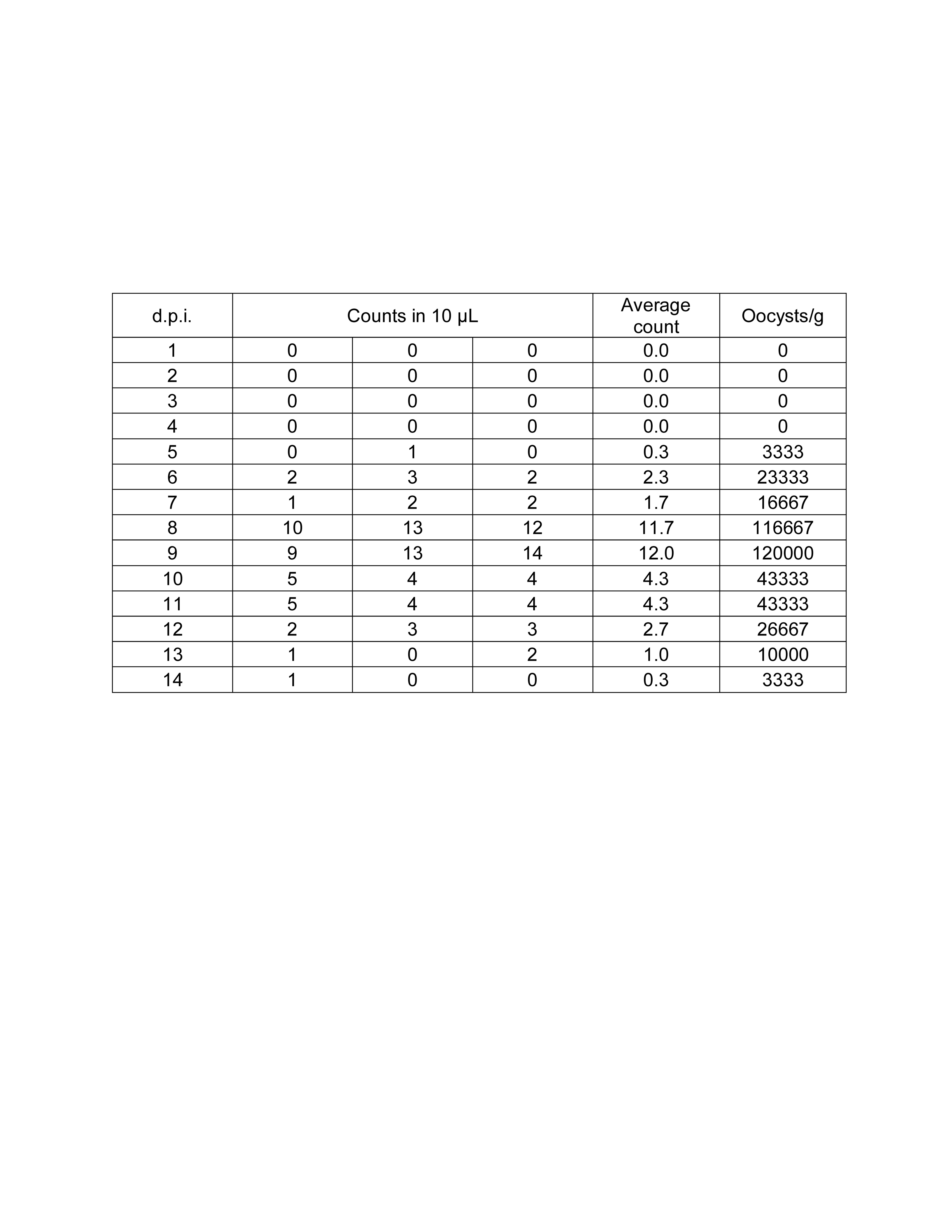

Supplement: S7 Data — Feces (0.5 g) was solubilized in 500 μL of saline, and then 10 μL of this suspension was counted in a hemocytometer. The values in the table are number of oocysts per sample. Each column of the table is an independent experiment, three biological replicates. The average counts per d.p.i. were used to calculate the number of oocysts/gram of feces. d.p.i., day post infection. (TIF) [file pbio.3000364.s010.tif]
