# Supplementary material for: Intestinal delta-6-desaturase activity determines host range for Toxoplasma sexual reproduction
Source: PLoS Biol. 2019 Aug 20;17(8):e3000364. doi: 10.1371/journal.pbio.3000364 (PMC6701743; doi:10.1371/journal.pbio.3000364)
Supplement: Attachment — Submitted filename: PLoS BioReferee.docx [file pbio.3000364.s011.docx]

Reviewer remarks:

Reviewer #1: Summary
The work described in this manuscript is truly transformative as it not only identifies the trigger of the Toxoplasma sexual cycle and why this only occurs in cats, it continues to fulfil Koch’s postulates by overcoming this species barrier by demonstrating the completion of the cycle in mice (and largely in vitro as well) by mimicking the cat environment. The work is also transformative in its approach as this is the first application of cat intestinal organoids to Toxoplasma. Besides the major biological breakthrough, these insights are relevant toward developing sexual recombination experiments in Toxoplasma (a tool that has been cumbersome due to its poor efficiency and the need of cats) as well as toward developing control strategies of cats shedding oocysts, which is a responsible for a large portion of human infections with Toxoplasma. In addition, the work is timely in light of the recent sudden and politically motivated suspension of the use of cats at the USDA, which supplied the majority of the Toxoplasma field with Toxoplasma oocysts. This work is therefore relevant for a very wide audience of microbiologists, infectious disease specialists, clinicians, epidemiologists and basic parasitology researchers. By fulfilling Koch’s postulates the paper per definition is very rigorous. In summary, a through breakthrough manuscript well described and executed. Only one minor comment as described below.
 
Specific comments.
Supplementary information; No Fig S2, but a fig 6. This is likely a mix up. Legend says “strained” instead of “stained” for DBA staining.

**Thank you for catching that reformatting error and typo.**

P7 line 13: in the sentence is referred to sero conversion, however, no data supporting this statement are shown in neither Fig 6e nor S2.

**We agree that the sero conversion data needs to be shown so we added it as supplemental figure 2.**

Reviewer #2 (Felix Yarovinsky, signed review): In this groundbreaking work the authors succeeded in answering a major biological question in the field of parasite biology.  It was a mystery why the sexual cycle of Toxoplasma gondii is limited to felines, while the asexual life cycle can happen in any warm-blooded mammal. Using an elegant in vitro system based on the intestinal organoids, the authors identified that linoleic acid is essential for the expression of markers associated with the sexual cycle of the parasite.  The authors provide the detailed biochemical explanation for this requirement.  It appears that cats are deficient in delta-6-desaturase, the rate‐limiting step for the conversion of linoleic acid to arachidonic acid. As a result, linoleic acid is the dominant fatty acid in cat serum. This explains why the felines have an increased level of linoleic acid.  
The authors next validated in vitro observation with mice that were fed with linoleic acid and delta-6-desaturase inhibitor.  This treatment results in the full sexual cycle of the parasite in the murine intestine.  This is a truly remarkable discovery in biology, and while there might be minor technical gaps in the work, I believe an immediate publication would benefit the scientific community. This is a highly significant work that not only teach us new biology, but also has a major clinical significance since it provides a novel biochemical tool to deal with toxoplasmosis in cats that may eventually lead to the elimination of this parasite in cats.  

I only have two minor comments 

1.      In the following statement ‘After 5 days of infection, we found that the addition of linoleic acid but not oleic acid caused approximately 35% of the T. gondii to express both merozoite stage markers (Fig. 2a)’ the authors forgot to acknowledge the data shown in Fig 1d-1g as an additional experimental support in the above-mentioned statement.

**Yes, we agree so we added reference to Fig 1d-g in that sentence.**

Figure 2, Y- axis:  the labeling needs to be more specific, as ‘%GRA11B and BRP1 positive’ is confusing.

**We agree so we changed this y-axis to read “% vacuoles GRA11B/BRP1 double positive”.**

Reviewer #3: Although it has been known for decades that felids are the definitive host for sexual replication of the intracellular protozoan parasite Toxoplasma gondii, the molecular underpinnings of this remarkable host specificity for parasite mating has remained an important unresolved mystery. The current work compellingly establishes that an excess linoleic acid drives sexual differentiation in culture and in mice, thus establishing for the first time in vitro and alternative in vivo models for T. gondii sexual development. The work represents a key breakthrough that will permit further dissection of sexual development, accelerate classical genetic linkage mapping of important traits, and reduce the dependence on using cats as an experimental model, among other avenues. The authors provide thorough qualitative and quantitative data that is appropriately interpreted and supportive of the conclusions. The following minor considerations should be addressed.

Line 21. Delete one of the “withs”

**Thanks for pointing out that we had two withs in line 21, we deleted the extra.**

Page 8, line 5-7. This statement should be softened from “likely” given that no data is presented to support it. It is suggested that the authors rephrase it to e.g., It is possible that the multiple…

**Thank you for pointing out this over statement. We agree so we changed the “likely” to “might”.**
